# The Complete Genome of a New Betabaculovirus from *Clostera anastomosis*

**DOI:** 10.1371/journal.pone.0132792

**Published:** 2015-07-13

**Authors:** Feifei Yin, Zheng Zhu, Xiaoping Liu, Dianhai Hou, Jun Wang, Lei Zhang, Manli Wang, Zheng Kou, Hualin Wang, Fei Deng, Zhihong Hu

**Affiliations:** 1 State Key Laboratory of Virology and China Center for Virus Culture Collection, Wuhan Institute of Virology, Chinese Academy of Sciences, Wuhan, 430071, PR China; 2 School of Tropical and Laboratory Medicine, Hainan Medical University, Haikou, 571101, PR China; University of Alabama at Birmingham, UNITED STATES

## Abstract

*Clostera anastomosis* (Lepidoptera: Notodontidae) is a defoliating forest insect pest. Clostera anastomosis granulovirus-B (ClasGV-B) belonging to the genus *Betabaculovirus* of family *Baculoviridae* has been used for biological control of the pest. Here we reported the full genome sequence of ClasGV-B and compared it to other previously sequenced baculoviruses. The circular double-stranded DNA genome is 107,439 bp in length, with a G+C content of 37.8% and contains 123 open reading frames (ORFs) representing 93% of the genome. ClasGV-B contains 37 baculovirus core genes, 25 lepidopteran baculovirus specific genes, 19 betabaculovirus specific genes, 39 other genes with homologues to baculoviruses and 3 ORFs unique to ClasGV-B. *Hrs* appear to be absent from the ClasGV-B genome, however, two non-*hr* repeats were found. Phylogenetic tree based on 37 core genes from 73 baculovirus genomes placed ClasGV-B in the clade b of betabaculoviruses and was most closely related to Erinnyis ello GV (ErelGV). The gene arrangement of ClasGV-B also shared the strongest collinearity with ErelGV but differed from Clostera anachoreta GV (ClanGV), Clostera anastomosis GV-A (ClasGV-A, previously also called CaLGV) and Epinotia aporema GV (EpapGV) with a 20 kb inversion. ClasGV-B genome contains three copies of polyhedron envelope protein gene (*pep*) and phylogenetic tree divides the PEPs of betabaculoviruses into three major clades: PEP-1, PEP-2 and PEP/P10. ClasGV-B also contains three homologues of P10 which all harbor an N-terminal coiled-coil domain and a C-terminal basic sequence. ClasGV-B encodes three fibroblast growth factor (FGF) homologues which are conserved in all sequenced betabaculoviruses. Phylogenetic analysis placed these three FGFs into different groups and suggested that the FGFs were evolved at the early stage of the betabaculovirus expansion. ClasGV-B is different from previously reported ClasGV-A and ClanGV isolated from *Notodontidae* in sequence and gene arrangement, indicating the virus is a new notodontid betabaculovirus.

## Introduction

Baculoviruses are invertebrate-specific viruses with circular, double-stranded DNA genomes ranging in size from 80–180 kb [1]. To date, more than 600 baculoviruses have been described and the family *Baculoviridae* is classified into four genera: *Alphabaculovirus* and *Betabaculovirus* which are lepidopteran-specific nucleopolyhedroviruses (NPVs) and granuloviruses (GVs), respectively, and, *Gammabaculovirus* and *Deltabaculovirus* encompassing NPVs that infect hymenopteran and dipteran insects, respectively [2]. Whereas NPVs occur in a number of insect orders, GVs are limited to the order Lepidoptera [3]. It is suggested that betabaculoviruses evolved earlier than the alphabaculoviruses [4]. Most betabaculoviruses exhibit a relatively narrow host range and their tissue tropism varies [3]. Based on tissue tropism, betabaculoviruses were subdivided into three types [5]. Type 1 betabaculoviruses, also known as slow-killing GVs, infect the midgut epithelium and fat body tissue. Representative member of the type is Xestia c-nigrum GV (XecnGV) and Helicoverpa armigera GV (HearGV) [6, 7]. The tissue tropism of type 2 betabaculoviruses, known as fast-killing GVs, is similar to typical lepidopteran NPVs that replicate in most of the host’s major tissues, and usually resulting in a fast speed of killing. Members belonging to this category include Plutella xylostella GV (PlxyGV), Cydia pomonella GV (CpGV) and Epinotia aporema GV (EpapGV) [8–10]. Type 3 betabaculoviruses are characterized by an infection restricted to the midgut epithelium. The only member of type 3 is Harrisina brillians granulovirus (HabrGV) and the virus has not been completely sequenced [11]. Phylogenetic analysis based on highly conserved genes of betabaculoviruses suggests that these different types of pathogenesis do not have monophyletic origins [12].

*Clostera anastomosis* (Lepidoptera: Notodontidae), distributed mainly in the Palearctic ecozone, is a defoliating forest insect pest considered to be the most important insect pest of poplar, willow and birch, reducing the regeneration and ornamental value of these trees [13]. Although chemical insecticides have been developed for the control of *C*. *anastomosis*, environmentally benign biological pesticides are needed to reduce the development of resistance by the insect and environmental pollution [14]. Baculoviruses have a long history of being used as biological insecticide because of the high infectivity and safety to the environment. Over 50 baculovirus insecticides have been used worldwide against various insect pests [15]. Several betabaculoviruses were isolated from diseased *C*. *anastomosis* from different areas in China and some of them had been used to control the pest [13, 16, 17].

The virus used in this paper is a highly pathogenic granulovirus isolated from a diseased *C*. *anastomosis* collected in Hunan province in China [18]. Previously a betabaculovirus has been isolated from *C*. *anachoreta* and was properly designated as ClanGV [19]. In addition, a betabaculovirus from *C*. *anastomosis* was fully sequenced and named as CaLGV [14] to distinguish it from ClanGV. Since CaLGV did not follow the convention in generating abbreviations for baculovirus, we suggest to rename CaLGV to ClasGV-A, and the virus reported here as ClasGV-B. The ClasGV-B was characterized as a type 2 “fast-killing GV” with a strong toxicity to the *C*. *anastomosis* larva suggesting potential applications for controlling the pest [13, 18]. To further characterize ClasGV-B, here we report the complete genome sequence of ClasGV-B and its gene organization in comparison with that of the other baculovirus genomes. The results showed that it is a new betabaculovirus isolated from *C*. *anastomosis* with different genome content and arrangement from that of ClasGV-A.

## Results and Discussion

### Sequencing and characteristics of the ClasGV-B genome

**Fig 1:**
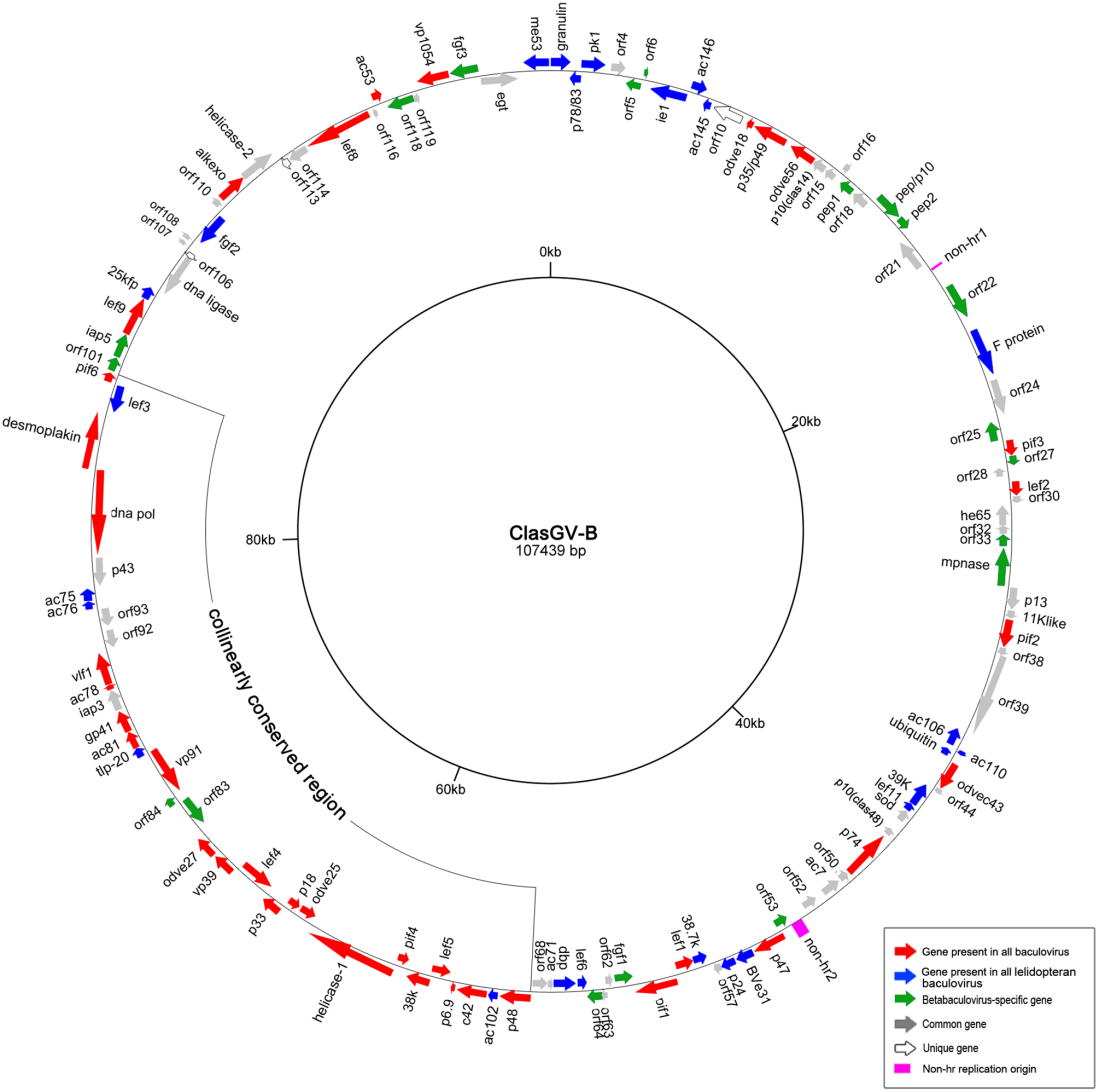
Circular map of ClasGV-B genome. ORFs and transcription direction are indicated as arrows. Core genes were indicated by red arrows, genes shared by all lepidopteran baculoviruses were indicated by blue arrows, betabaculovirus-specific genes were indicated by green arrows, genes with homologues to other baculoviruses were indicated by grey arrows, unique genes were indicated by open arrows and non-*hrs* were indicated by pink squares. The highly collinearly conserved region found in alpha- and betabaculoviruses was shown. Genome position was shown by a 20 kb scale in the inner circle.

Approximately 110 times coverage of the ClasGV-B genome was achieved from 11,950,277 nt of data generated by 454 pyrosequencing. The genome is 107,439 nt in length with a G+C content of 37.8%. One hundred twenty-two methionine-initiated ORFs of 150 nt or more in length with minimal overlap were indentified (Fig 1 and S1 Table). In addition, ClasGV-B ORF116 (46 aa in length) was also considered a putative ORF as it showed homology to ORFs of other baculoviruses. The coding regions covered 93% of the genome and the *granulin* gene was designated *orf1* with its first nt of the start codon as nt 1 of the genome. Sixty-three ORFs were clockwise directed and sixty ORFs were counterclockwise directed in respect to the transcriptional orientation of the granulin gene (Fig 1). Twenty-nine overlaps were observed between adjacent ORFs, among which, overlap of more than 75 bp were found between *orf37 (per os infectivity factor 2*, *pif-2)* and *orf38* (77 bp), *orf53* and *orf54* (*p47*) (105 bp), *orf86* (*telokin-like protein-20*, *tlp-20*) and *orf87* (122 bp), *orf90* and *orf91* (*very late factor 1*, *vlf-1*) (80 bp), *orf111* (*alkaline nuclease*, *alk-exo*) and *orf112* (*helicase-2*) (83 bp).

### Overall gene content of the ClasGV-B genome

Previously, 37 core genes of baculovirus were identified based on the analysis of 58 completely sequenced genomes [20]. All of these 37 core genes were identified in ClasGV-B genomes (showing red in Fig 1).

Baculoviruses within the same taxonomic subgroup were found to have an additional set of shared genes beside the core genes [21]. So far, baculoviruses infecting lepidopteran hosts have a total number of 25 shared genes [20–22]. All these genes were identified in ClasGV-B genome (showed in blue in Fig 1): *granulin* (*orf1*), *p78/83* (*orf2*), *protein kinase 1* (*pk-1*, *orf3*), *immediate early protein 1* (*ie-1*, *orf7*), *ac146* (*orf8)*, *ac145* (*orf9*), *envelope fusion protein* (*F protein*, *orf23*), *ac106* (*orf40*), *ac110* (*orf41*), *ubiquitin* (*orf42*), *39K* (*orf45*), *late expression factor 11* (*lef-11*, *orf46*), *bv-e31* (*orf55*), *p24* (*orf56*), *38*.*7K* (*orf58*), *lef-6* (*orf65*), *DNA binding protein* (*dbp*, *orf66*), *ac102* (*orf70*), *tlp-20* (*orf86*), *ac76* (*orf94*), *ac75* (*orf95*), *lef-3* (*orf99*), *25k-fp* (*orf104*), *fibroblast growth factor-2* (*fgf-2*, *orf109*), *major envelope protein 53* (*me53*, *orf123*).

Betabaculovirus-specific genes are considered important genomic criteria distinguishing GVs from NPVs, and the number of betabaculovirus-specific genes has changed as more genomes were sequenced. To date, a set of 19 genes are shared by all betabaculoviruses but were not identified in alpha-, gamma- or deltabaculovirus genomes [23]. All the 19 genes were identified in ClasGV-B genome (showed in green in Fig 1): *orf5*, *orf6*, *polyhedron envelope protein 1* (*pep-1*, *orf17*), *pep/p10* (*orf19*), *pep-2* (*orf20*), *orf22*, *orf25*, *orf27*, *orf33*, *matrix metalloprotease* (*mp-nase*, *orf34*), *orf53*, *fgf-1* (*orf61*), *orf64*, *orf83*, *orf84*, *orf101*, *inhibitor of apoptosis 5* (*iap-5*, *orf102*), *orf118*, *fgf-3* (*orf121*).

Apart from the above genes, 39 ORFs were found to have homologues in other baculoviruses (Table 1 and Fig 1), and 3 ORFs (*orf10*, *orf106* and *orf113*) are, so far, unique to ClasGV-B (Fig 1).

**Table 1:** ClasGV-B genes grouped according to function.

	Genes present in ClasGV-B*	Genes missing in ClasGV-B
Replication	*ie-1*(*orf7*), ***lef-2*(** ***orf*** ***29*)**, ***lef-1*(** ***orf*** ***59*)**, *dbp*(*orf66*), ***helicase-1*(** ***orf*** ***76*)**, ***dna pol*(** ***orf*** ***97*)**, ***lef-3*(** ***orf*** ***99*)**, *dna ligase*(*orf105*), *helicase-2*(*orf112*), *me53* (*orf123*)	*rr1*, *rr2*, *dUTPase*
Transcription	*39k*(*orf45*), *lef-11*(*orf46*), ***p47*(*orf54*)**, *lef-6*(*orf65*), ***lef-5*(*orf73*)**, ***lef-4*(*orf80*)**, ***lef-9*(*orf103*)**, ***lef-8*(*orf115*)**, ***vlf-1*(*orf91*)**	*ie-0*, *ie-2*, *pe-38*,*lef-10*
Structure	*granulin*(*orf1*), *pk-1*(*orf3*), ***odv-e18*(*orf11*)**, *p10*(*orf14*, *orf48*), *pep-1*(*orf17*), *pep/p10*(*orf19*), *pep-2*(*orf20*), *f protein*(*orf23*), *mp-nase*(*orf34*), ***odv-ec43*(*orf43*)**, *BV-e31*(*orf55*), *p24*(*orf56*), ***p48*(*orf69*)**, *c42*(*orf71*), ***p6*.*9*(*orf72*)**, *odv-e25*(*orf77*), ***vp39*(*orf81*)**, ***odv-e27*(*orf82*)**, ***vp91*(*orf85*)**, ***gp41*(*orf88*)**, *25k-fp(orf104)*, *orf116*, ***orf117***, *orf118*, *orf119*, ***vp1054*(*orf120*)**	*odv-e66*
PIFs or genes related to oral infection	***p74*(*orf49*)**, ***pif-1*(*orf60*)**, ***pif-2*(*orf37*)**, ***pif-3*(*orf26*)**, ***pif-4*(*orf75*)**, ***odv-e56/pif-5* (*orf13*)**, ***odv-nc42/pif-6*(*orf100*)**	*odv-e66*, *enhancin*
Auxiliary gene	***p35/49*(*orf12*)**, *mp-nase*(*orf34*) *ubiquitin*(*orf42*), *sod*(*orf47*) *fgf-1*(*orf61*), *iap-3*(*orf89*), *iap-5*(*orf102*), *fgf-2*(*orf109*), ***alk-exo*(*orf111*)**, *fgf-3*(*orf121*), *egt(orf122)*	*bro*, *chitinase*, *cathepsin*, *ctl*, *gp37*, *lef-10*
Unique	*orf10*, *orf106*, *orf113*	

*Core genes are shown in bold and lepidopteran baculovirus specific genes are underlined.

### Comparisons to other baculoviruses

**Fig 2:**
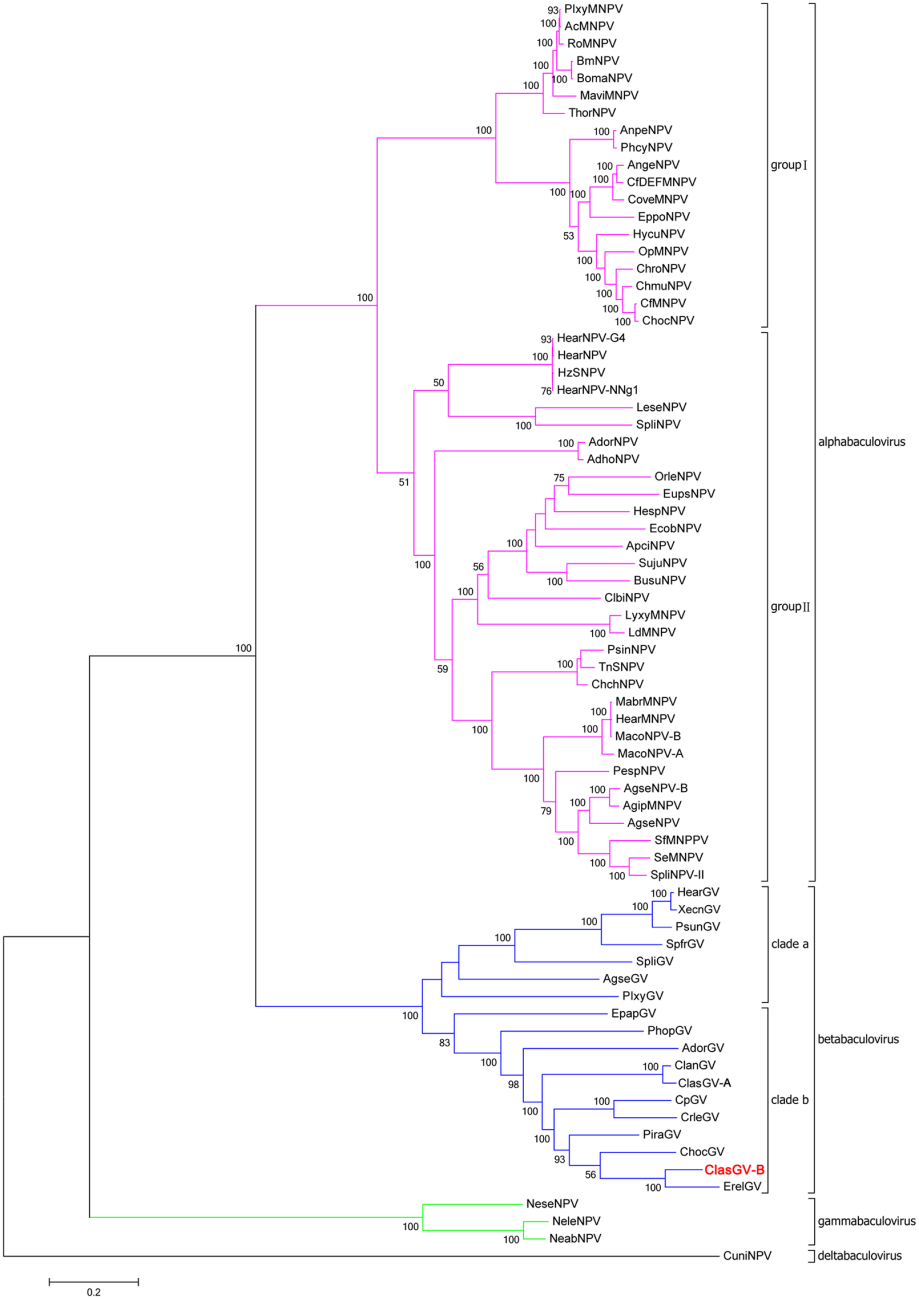
Phylogenetic tree based on concatenation of 37 conserved core proteins from 73 baculovirus genomes. Phylogenetic analysis was performed using the Maximum Likelihood method and bootstrap analysis for 1000 replicates was performed. Only bootstrap values over 50% were shown. The bar lengths are proportional to the distances of the baculoviruses.

It has been demonstrated that phylogenetic trees generated from the concatenation of shared genes are more reliable than that from the individual genes [24]. A phylogenetic tree derived from concatamers of the aa sequences of the 37 core genes from 73 fully sequenced baculoviruses is shown in Fig 2. Previous phylogenetic analysis of baculovirus revealed that the betabaculoviruses tend to cluster into two clades [25]. Clade “a” includes five sequenced betabaculoviruses from noctuid hosts [Agrotis segetum GV (AgseGV), Spodoptera litura GV (SpliGV), Pseudaletia unipuncta GV (PsunGV), HearGV and XecnGV], as well as PlxyGV whose host is a plutellid [10, 25]. The members of the clade “b” includes five tortricid GVs (EpapGV, Adoxophyes orana GV (AdorGV), CpGV, Cryptophlebia leucotreta GV (CrleGV) and Choristoneura occidentalis GV (ChocGV)), as well as Pieris rapae GV (PiraGV) isolated from *Pieridae*,Phthorimaea operculella GV (PhopGV) from *Gelechiidae*, Erinnyis ello GV (ErelGV) from *Sphingidae*, as well as ClanGV and ClasGV-A from *Notodontidae* [25]. In the phylogenetic tree built in this study, ClasGV-B is grouped in clade b. However, bootstrap analysis showed that it is not most closely related to ClanGV and ClasGV-A, which are also isolated from *Notodontidae*, but is more closely related to ErelGV from *Sphingidae* (Fig 2). The overall nucleotide identity of ClasGV-B genome to that of ErelGV, ClanGV and ClasGV-A is 75%, 67% and 82%, respectively.

**Fig 3:**
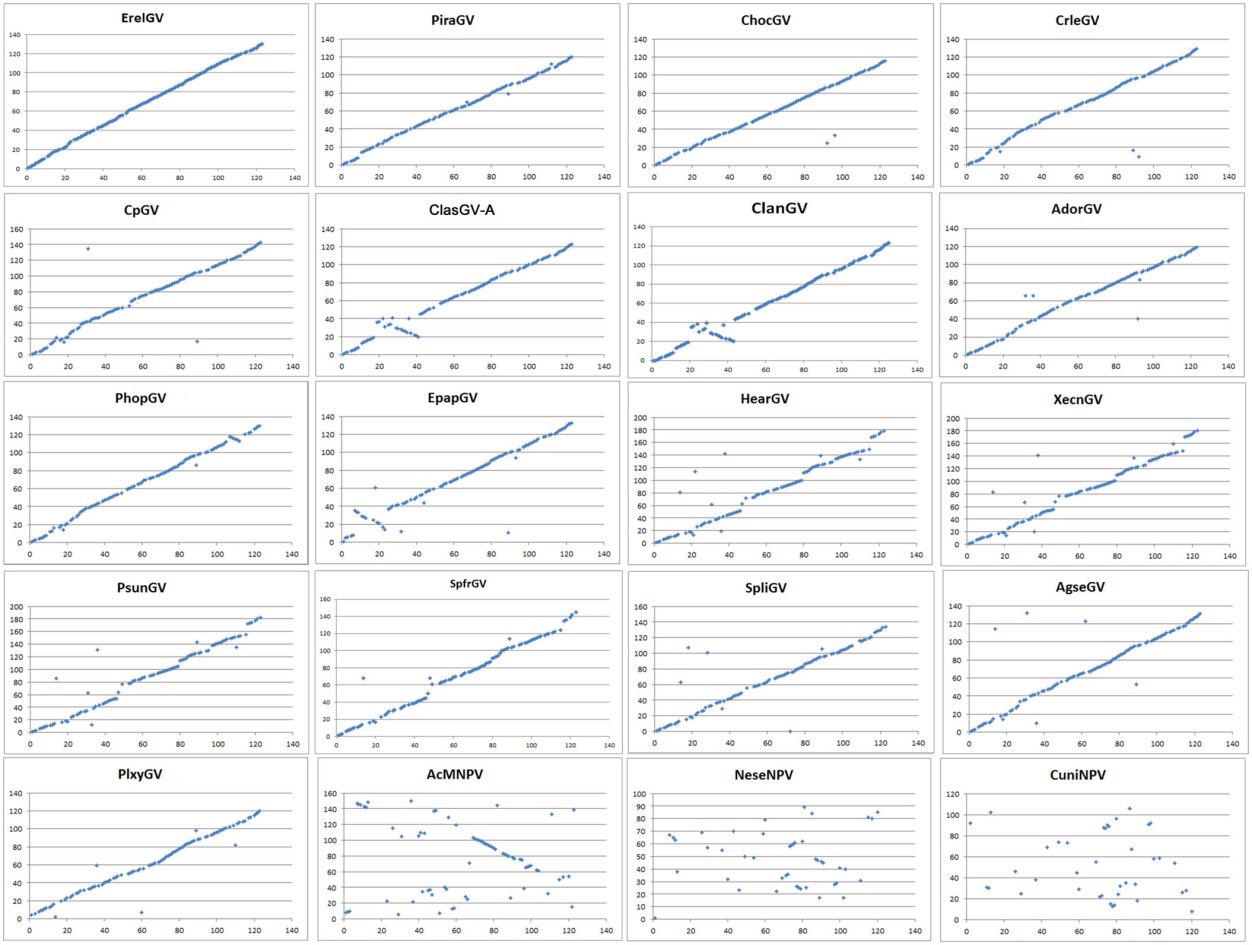
Gene Parity Plot analyses of ClasGV-B with 17 other sequenced betabaculoviruses, AcMNPV, NeseNPV and CuniNPV. Homologous genes were plotted based on their relative location in the genomes. The x axis represents ORFs from ClasGV-B and y axis represents ORFs from the virus shown in the title of each panel.

Comparative analysis revealed that gene arrangements in baculoviruse genomes can serve as phylogenetic markers, with viruses sharing a higher degree of gene collinearity are more closely related [4]. Strong gene collinearity is observed in sequenced betabaculoviruses genomes [26]. In this study, gene order of ClasGV-B was compared to all other sequenced betabaculoviruses and to Autographa californica MNPV (AcMNPV), Neodiprion sertifer NPV (NeseNPV) and Culex nigripalpus NPV (CuniNPV), the representatives of alpha-, gamma- and deltabaculoviruses, using Gene Parity Plot [27]. As expected, gene order of ClasGV-B is substantially collinear with other betabaculoviruses but its gene arrangement is significantly different from that of AcMNPV, NeseNPV and CuniNPV (Fig 3). Among betabaculoviruses, ClasGV-B shared the strongest collinearity with ErelGV, ChocGV, PiraGV and CpGV but differed from ClanGV, ClasGV-A and EpapGV with a 20 kb inversion (Fig 3).

The highly collinearly conserved region found in alpha- and betabaculoviruses [22] was also observed in ClasGV-B (Fig 1). In this region of ClasGV-B, besides the reported 20 core genes and 5 lepidopteran conserved genes [22], it also contains another core gene (*odv-e27*), two belabaculovirus specific genes (*orf83* and *orf84*) and four other genes (*iap-3*, *orf92*, *orf93* and *p43*) (Fig 1).

### Genes involved in DNA replication and transcription

Many genes involved in DNA replication and transcription have been identified in sequenced baculovirus genomes. Homologues of all the six genes essential for DNA replication are present in ClasGV-B genome; *lef-1* (*orf59*), *lef-2* (*orf29*), *lef-3* (*orf99*), *dna polymerase* (*dna pol*, *orf97*), *helicase-1* (*orf76*), and *ie-1* (*orf7*) (Table 1). Other genes implicated in DNA replication and found in ClasGV-B are *dna ligase* (*orf105*), *dbp* (*orf66*) and *me53* (*orf123*) (Table 1). ClasGV-B did not encode *lef-7* or *lef-12* which typically found in group I alphabaculoviruses. Similar to most betabaculoviruses and four group II alphabaculoviruses (Lymantria dispar MNPV (LdMNPV), Lymantria xylina MNPV (LyxyMNPV), Mamestraconfigurata NPV-B (MacoNPV-B) and Orgyia leucostigma NPV (OrleNPV)), ClasGV-B encodes both a *dna ligase* (*orf105*) and a second *helicase* (*helicase-2*, *orf112*) [4]. *Helicase-2* is a member of the *helicase* superfamily I and is different from *helicase-1* which is longer and has low homology to *helicase* genes from other organisms [4, 21]. The function of DNA ligase and Helicase-2 is proposed to be in DNA repair and recombination [28].

Genes coding enzymes involved in nucleotide metabolism such as large (*ribonucleotide reductase 1*, *rr1*) and small (*rr2*) subunits of ribonucleotide reductase, and deoxyuridyl triphosphate (*dUTPase*) were absent from the ClasGV-B genome. These genes were first identified in *Orgyia pseudotsugata* MNPV (OpMNPV) and found in some alpha- and betabaculoviruses [29]. They constitute the DNA repair system of baculoviruses with ribonucleotide reductase catalyzes the reduction of host cell rNTPs to dNTPs while dUTPase prevents the incorporation of uracyl into DNA [30].

The function of baculovirus genomes is organized into a temporarily regulated cascade of gene expression classified as early, late and very late genes [21]. Early genes are transcribed by host RNA polymerase II, but late and very late genes are transcribed by a viral encoded RNA polymerase [31]. ClasGV-B encodes all the RNA polymerase subunits: *lef-4* (*orf80*), *lef-9* (*orf103*), *lef-8* (*orf115*), *lef-5* (*orf73*), *p47* (*orf54*) and *vlf-1* (*orf91*) (Table 1). Four genes, *ie-0*, *ie-1*, *ie-2* and *pe38*, have been reported to transactivate transcription of early baculoviral genes [32]. Of these genes, only *ie-1* (*orf7*), found in all lepidopteran baculoviruses, is present in ClasGV-B genome [33]. Both *pe38* and *ie-2* are absent from all sequenced group II alphabaculoviruses and betabaculoviruses except for CpGV, CrleGV, PhopGV, and PiraGV that contain a *pe38* gene. Additional genes involved in late gene transcription found in all lepidopteran baculovirus were also identified in ClasGV-B including *lef-6* (*orf65*), *lef-11* (*orf46*), *39k* (*orf45*) and *pk-1* (*orf3*). *Lef-10*, which involved in late gene transcription and detected in many lepidopteran baculoviruses, was absent from ClasGV-B genome.

### Structural genes

ClasGV-B encodes the following core genes for baculovirus structure: *p6*.*9* (*orf72*), *vp39* (*orf81*), *vp1054* (*orf120*), *vp91* (*orf85*), *gp41* (*orf88*), *odv-ec43* (*orf43*), *odv-e18* (*orf11*). Other structural genes presented in ClasGV-B that are lepidopteran-specific include *granulin* (*orf1*), *pk-1* (*orf3*), *25k-fp* (*orf104*), *odv-e25* (*orf77*) and *c42* (*orf71*).

Baculoviruses encode two distinct envelope fusion proteins, GP64 and F protein. GP64 is essential for the budded virus of group I alphabaculoviruses [34]. In contrast, no *g64* homologue was identified in group II alpha-, beta-, and deltabaculoviruses, but another envelope fusion protein F was proved to be a functional envelope fusion protein [35, 36]. Gammabaculoviruses do not contain either protein. There is no sequence homology between *gp64* and *F* gene, but F proteins from group II alpha- and betabaculoviruses can replace the function of AcMNPV GP64 with the exception of PlxyGV F protein [37, 38]. The identity of ClasGV-B F protein (ORF23) with other sequenced betabaculovirus F proteins ranged from 73% with Erel28 to 33% with Psun26.

**Fig 4:**
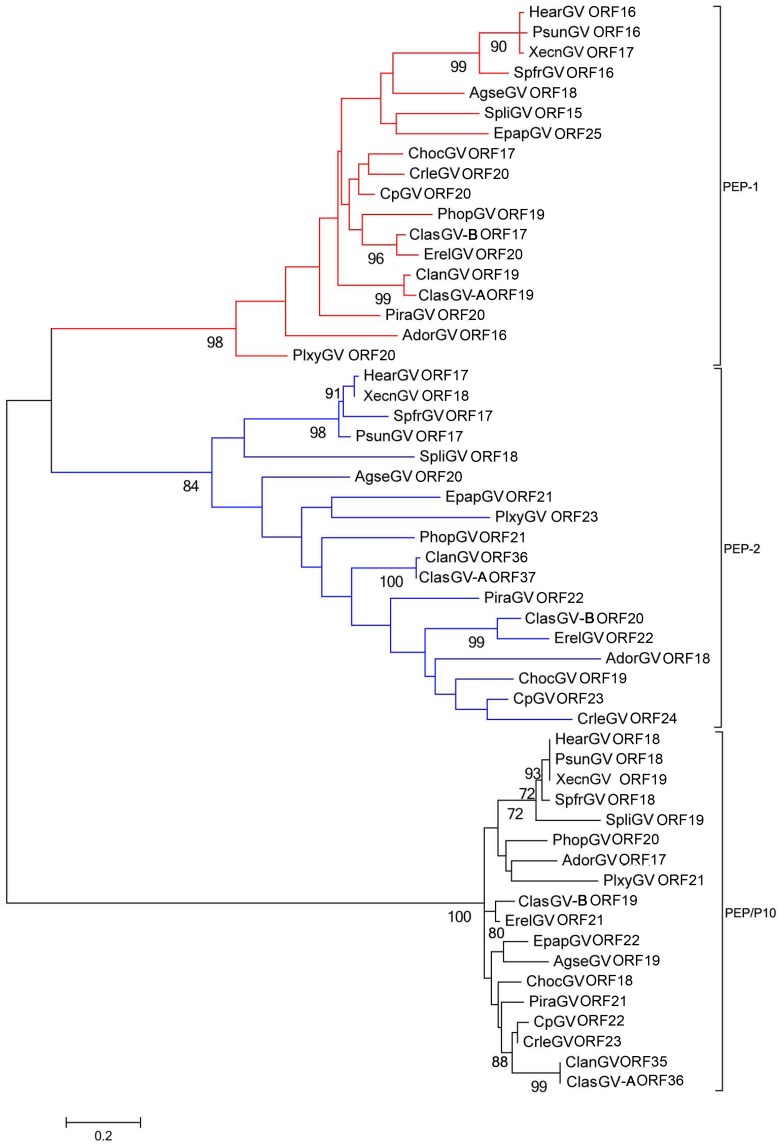
Phylogenetic analysis of PEP-1, PEP-2 and PEP/P10 from all the sequenced betabaculoviruses. PEP-1, PEP-2 and PEP/P10 were indicated by red, blue and black branches respectively. The tree was constructed based on protein sequences using the Maximum Likelihood method and the bootstrap analysis for 1000 replicates was conducted. Only bootstrap values over 50% were shown. The bar lengths are proportional to the distances of gene.

As in other betabaculoviruses, ClasGV-B genome contains three copies (*orf17*, *orf19*, *orf20*) of *pep* gene in a similar location and all these genes share a relatively high amino acid identity, ranging from 37% to 72%. PEP is located on the surface of occlusion bodies (OBs) and functions as a stabilizing factor for polyhedron [39]. The PEP in alphabaculoviruses consists of a N-terminal domain (PEP-N), a C-terminal domain (PEP-C) and a second C-terminal domain (C2) [1]. A pervious study reported that there are two kinds of PEPs in betabaculoviruseses; 1) with PEP-N and C2 domains, but without PEP-C; and 2) with PEP-N and C2 domains, but PEP-C is replaced by P10-like sequence [1]. In ClasGV-B, PEP-1 (ORF17) and PEP-2 (ORF20) are without PEP-C and PEP/P10 (ORF19) is with P10 like sequence. A phylogenetic tree has been established by using PEPs from all the sequenced betabaculoviruses (Fig 4). The phylogenetic tree clearly divides the PEPs into three major clades: PEP-1, PEP-2 and PEP/P10. The result suggests that possibly *pep-1* gene and *pep-2* gene constitute a pair of paralogous genes and *pep/p10* originated separately from them and all the *pep* genes were captured in the early stage of betabaculovirus radiation.

**Fig 5:**
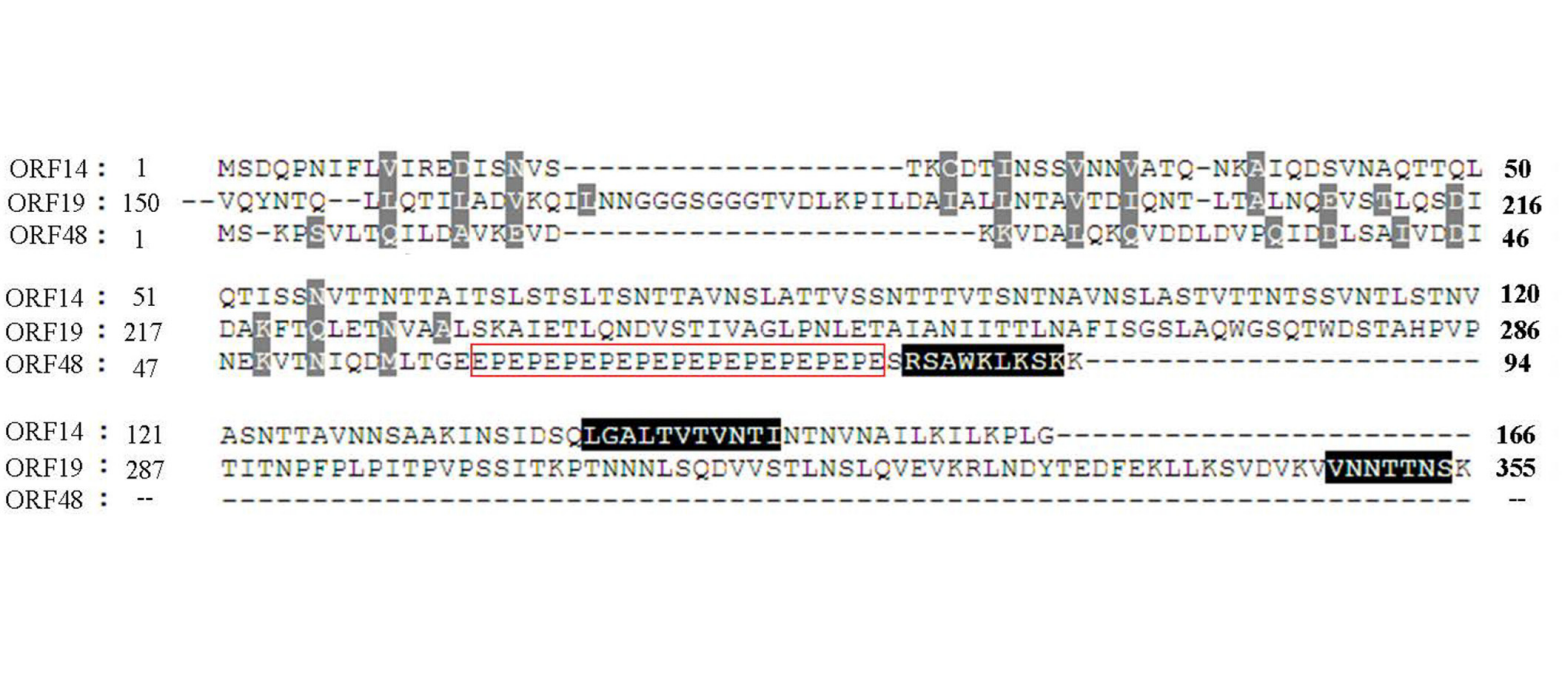
Alignment of ClasGV-B P10 proteins. Clustal W alignment of ClasGV-B P10 proteins is shown. The coiled-coil region was shaded in light grey, and the basic region in black, and the proline-rich region was indicated by a red frame. The positions of the aa in the proteins are indicated.

Three *p10* homologous were detected in ClasGV-B genome: *orf14*, *orf19* and *orf48*. P10 proteins are generally poorly conserved and are characterized by size differences in shared structural and functional domains [40]. All ClasGV-B P10s shared an N-terminal coiled-coil domain and a C-terminal basic sequence. Between the domains, ORF48 had a proline-glutamic acid (PE) repeat (Fig 5). Like in most betabaculoviruses, *orf19* encoded a fusion protein with PEP at the N-terminus and P10 at the C-terminus. It is well documented that P10 and the polyhedron envelope are closely associated for OB morphogenesis in NPVs [41]. The functionally associated P10 and PEP is conserved as a single protein in all of the fully sequenced betabaculoviruses except in PiraGV [1]. It remains elusive why betabaculoviruses contain three copies of PEPs and P10s in their genomes.

### Per os infectivity factors and other genes involved in oral infection

PIFs are proteins essential for oral infection of insect larvae [42]. So far 7 *pif* genes have been identified [43–45] and they are all conserved in ClasGV-B genome; *p74* (*orf49*), *pif-1* (*orf60*), *pif-2* (*orf37*), *pif-3* (*orf26*), *pif-4* (*orf75*), *odv-e56/pif-5* (*orf13*), and *odv-nc42/pif-6* (*orf100*) (Table 1).

Enhancin which is classified as metalloprotease is proposed to enhance oral infection by degrading the peritrophic matrix [46, 47]. *Enhancin* gene is absent in ClasGV-B but has been identified in the genomes of six betabaculoviruses including: HearGV, PsunGV, Trichoplusia ni GV (TnGV), XecnGV, AgseGV and Choristoneura fumiferana GV (ChfuGV) [48]. ODV-E66 is a chondrotinase which also disrupts the peritrophic matrix to facilitate oral infection [48]. *Odv-e66* gene is present in the genome of ClanGV and ClasGV-A [14] but no *odv-e66* homolog was found in ClasGV-B indicating again the difference of ClasGV-B to ClanGV and ClasGV-A.

### Auxiliary genes

**Fig 6:**
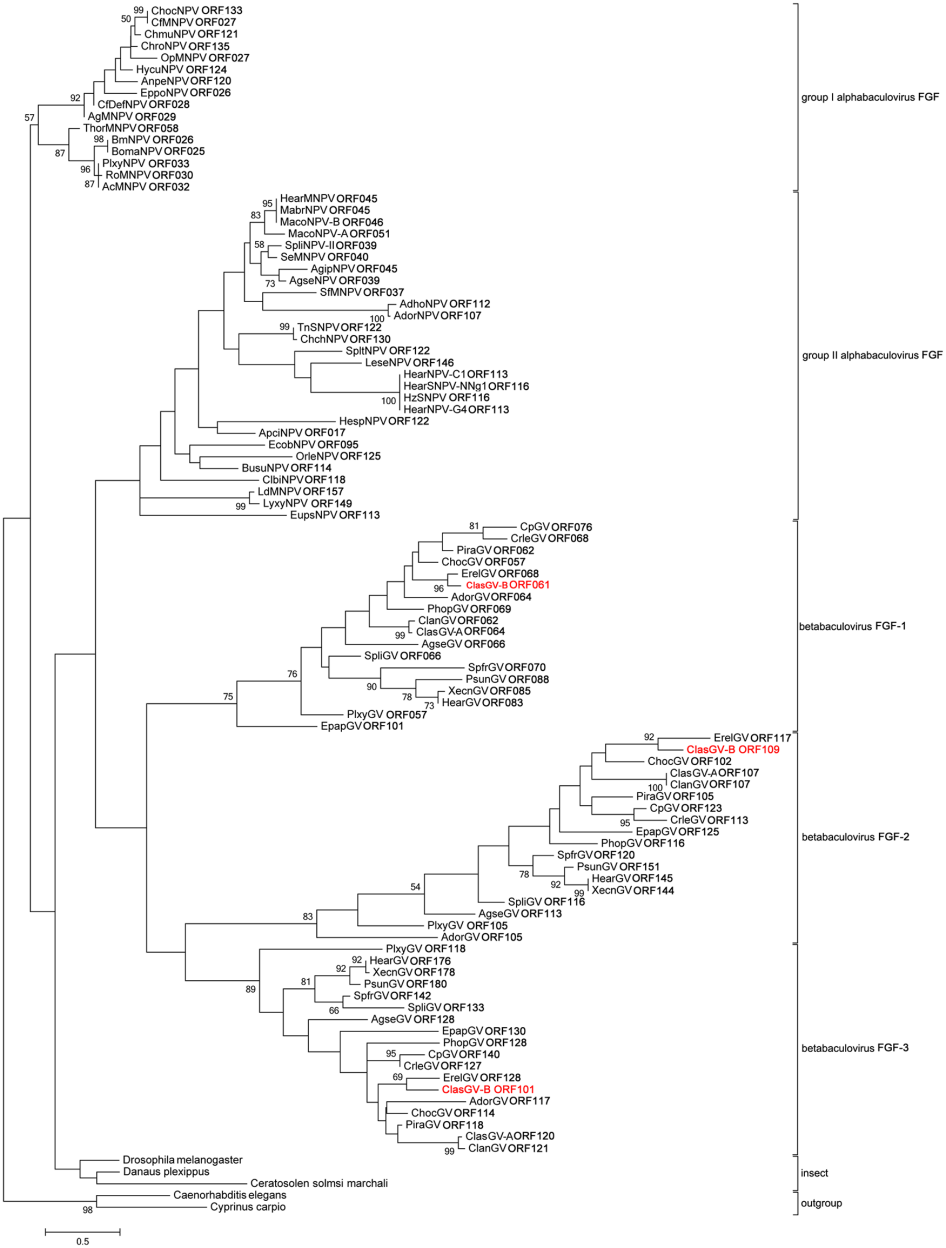
Phylogenetic analysis of FGF of all the sequenced baculoviruses. The tree was constructed based on protein sequences using the Maximum Likelihood method, the bootstrap analysis was done for 1000 replicates and only the bootstrap values over 50 were shown. The GenBank accession number of FGF of insects and out group FGFs: NP_732452.1 (*Drosophila melanogaster*), EHJ75405.1 (*Danaus plexippus*), XP_011496166.1 (*Ceratosolen solmsi* marchali), NP_498403.1 (*Caenorhabditis elegans*), AHM24043.1 (*Cyprinus carpio*).

Auxiliary genes are not essential for viral replication, but they may provide the virus selective advantages in nature [49]. ClasGV-B encodes three fibroblast growth factor homologues, FGF-1 (ORF61), FGF-2 (ORF109) and FGF-3 (ORF121). These three *fgfs* are conserved in all sequenced betabaculoviruses and homologues of *fgf* are also found in all alphabaculoviruses. FGF from alphabaculoviruses contains a predicted N-terminal signal peptide and a typical FGF superfamily central motif of ~ 120 aa essential for binding to an FGF receptor [50]. The function of FGF in group I alphabaculoviruses was characterized to accelerate host mortality by enhancing systemic infection after initial infection of midgut epithelium. Deletion of *fgf* in AcMNPV and Bombyx mori nucleopolyhedrovirus (BmNPV) resulted in delayed time of death of the infected larvae [51, 52]. A phylogenetic tree was built with FGFs of all the sequenced baculoviruses (Fig 6). FGFs of insects which were suggested to be closely related to baculovirus FGF [50] were included in the phylogenetic analysis and FGFs of *Caenorhabditis elegans* and *Cyprinus carpio* were used as the out group of the phylogenentic tree. The topology and bootstrap values of the phylogenetic tree suggested that baculoviral FGFs could be classified into 5 groups: groupIalphabaculovirus FGF, group II alphabaculovirus FGF, betabaculovirus FGF-1, betabaculovirus FGF-2, and betabaculovirus FGF-3 (Fig 6). In betabaculovirus groups, the general branches topology of the three FGF groups is similar to the branches of the betabaculovius evolutionary tree (Fig 2), implying that the FGFs were evolved at the early stage of the betabaculovirus expansion. It remains unclear as to why betabaculoviruses contain three copies of *fgfs* and whether they function differently during viral infection.

Baculoviruses contain two families of genes that suppress apoptosis triggered at the early stages of infection, *p35/p49* and *iap*. These are two different classes of genes with different mode of action. P35/P49 directly inhibit the activity of proteases, while IAPs act more upstream to prevent the activation of proteases [53]. Homologues of P35/P49 are present in some but not in all baculoviruses, whereas IAPs present in all sequenced baculoviruses. Based on the general structure and functional motifs, two baculovirus IAP repeats (BIRs) and one C-terminal zinc finger-like (RING) *Cys/his* motif, IAPs were divided into five groups, IAP-1 to IAP-5, but not all are active suppressors of apoptosis [54]. Homologues of *iap-5* were identified specifically in all sequenced betabaculoviruses. ClasGV-B possesses two *iap* genes, *iap-3* (*orf89*) and *iap-5* (*orf102*), and one *p35/p49* (*orf12*) gene.

ClasGV-B genome contains *ubiquitin* (*orf42*) and *superoxide dismutase* (*sod*, *orf47*), both of which are well conserved in baculoviruses. ClasGV-B shared a mean amino acid identity of 83% and 63% to the Ubiquitins and SODs from all other sequenced betabaculoviruses, respectively. The function of these genes in baculoviruses is still unknown.

*Cathepsin* and *chitinase* present in some betabaculoviruses and in most alphabaculoviruses are absent from ClasGV-B genome. These genes are related to the degradation of insect tissues and host liquefaction late in the infection, and thus facilitate horizontal spread of the virus [55, 56]. ClasGV-B encode *mp-nase* (*orf34*) gene which is a betabaculovirus-specific gene and proposed to be involved in basement membrane degradation to aid in systemic infection of BV [57].

Baculovirus repeated ORFs (*bro* genes) comprise a highly repetitive and conserved gene family whose function is not totally clear. One to sixteen copies of the *bro* gene present in some betabaculoviruses and in all sequenced alpha- and deltabaculoviruses [58]. ClasGV-B genome does not appear to contain a *bro* gene.

### Unique genes

Three ORFs (ORF10, ORF106 and ORF113) appear to be unique to ClasGV-B compared to the other baculoviruse ORFs. *Orf10* and *orf113* encode proteins of 387 aa and 119 aa long respectively and neither of the proteins have significant BlastP matches. *Orf106* encodes for a 61 aa long protein sharing a very low homology to the proteins of glycosyl hydrolases family 2 in *Porphyromonas sp*. (minimum E value = 3.4).

### Repeat regions

A common feature in most baculovirus genomes is the presence of homologous regions (*hr*s) interspersed throughout the genome. *Hr* has been found in all of the sequenced baculoviruses with the exception of *Trichoplusia ni* single NPV (TnSNPV), *Chrysodeixis chalcites* NPV (ChchNPV), AgseGV and *Buzura suppressaria* NPV (BusuNPV). The *hr*s act as enhancers of transcription of early genes and may serve as origins of replication (*ori*) and as sites of recombination [33]. *Hr*s are AT-rich and their structure varies widely in terms of length, sequence and copy number of tandem repeats that consists of an imperfect palindrome. Typical *hr* of NPVs consists of a 30 bp imperfect palindrome within direct repeats, whereas majority of *hr*s in betabaculoviruses are poorly conserved and often lack palindromes [59]. Conserved structure of *hr*s with palindromes were only found in betabaculoviruses of tortricids including CpGV, CrleGV, AdorGV, ChocGV and EpapGV, and in PhopGV, which infects host of the *Gelechiidae* family [10]. *Hr* is absent from the ClasGV-B genome.

**Fig 7:**
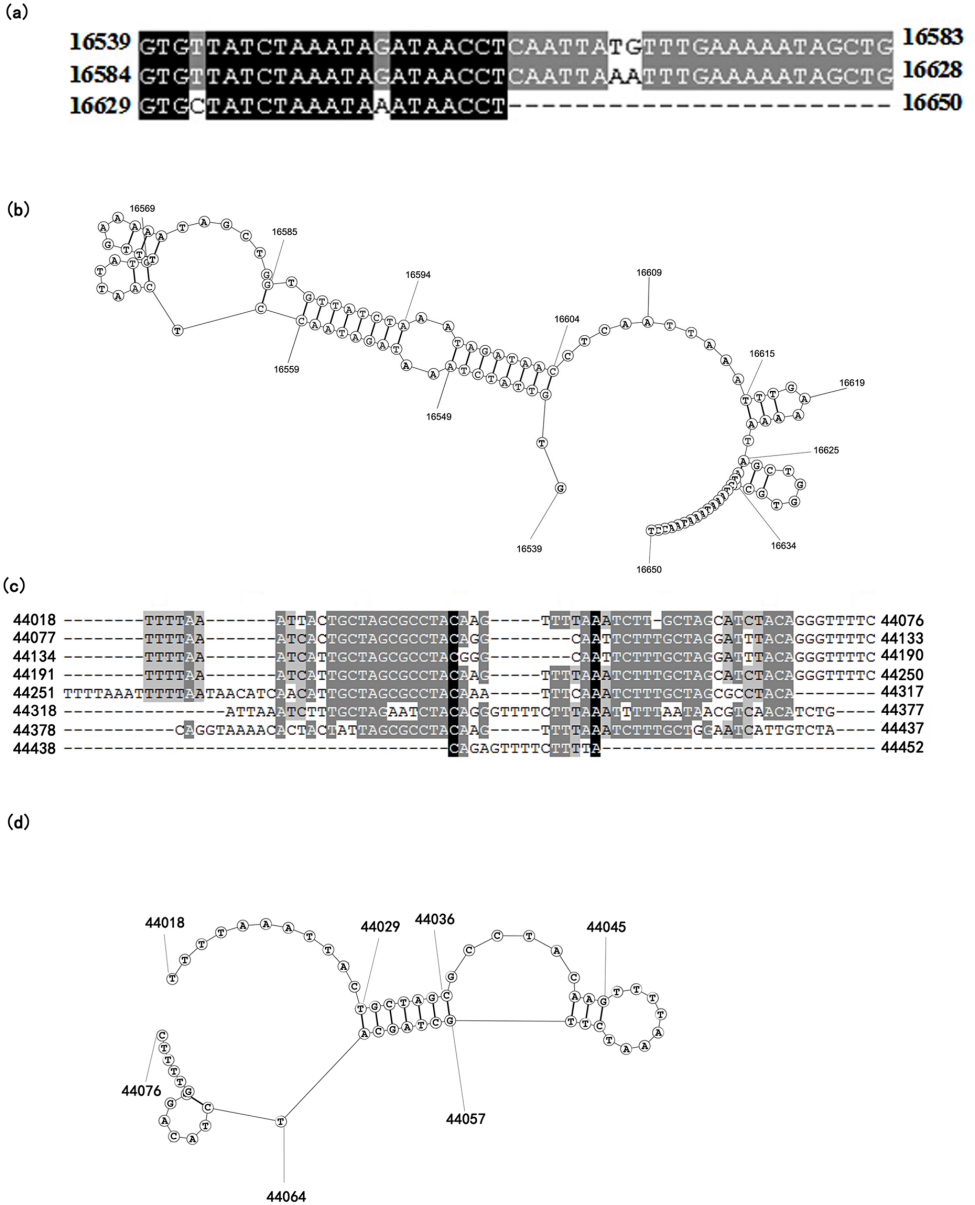
Alignments and predictions of the secondary structures of ClasGV-B non-hrs. Nucleotide alignments of non-*hr1* (a) and non-*hr2* (c) as well as predicted secondary structures of non-*hr1*(b) and non-*hr2* (d) were shown.

Some baculoviruses contain another type of putative *ori*, the so-called non-*hr ori*, which consists of palindromes and direct repeats neighboured by an AT-rich region. Two putative non-*hr*s (non-*hr*1 and non-*hr*2) were found in large intergenic spacers between *orf21* and *orf22*, and between *orf52* and *orf53*, respectively. Non-*hr*1 covered a 112 bp region with two direct large imperfect repeats and a truncated imperfect repeat (Fig 7a) while non-*hr*2 covered a 435 bp region with seven direct large imperfect repeats and a truncated imperfect repeat (Fig 7c). These regions are AT-rich (74% for non-*hr*1 and 66% for non-*hr*2) and contain palindromic sequences predicted to form a hairpin structure (Fig 7b and 7d). Large AT-rich intergenic spacers with repeated sequences were also found in CrleGV, CpGV, ChocGV and EpapGV. The 1.8 kb intergenic spacer of CrleGV located between *Crle26* and *Crle27* contains a 300 bp AT-rich region with direct repeats and short palindromes. *In vitro* analysis indicated that the short repeats concatenated during virus replication and are considered to serve as non-*hr* replication origin [1, 60]. The large region of repeated sequences identified in CpGV (between *Cp25* and *Cp27*), ChocGV (between *Choc36* and *choc37*) and EpapGV (between *Epap17* and *Epap19*) are also considered to be a non-*hr* replication origin [8, 10, 61]. Non-*hr*1 *i*n ClasGV-B is in approximately the same location with respect to the surrounding ORFs as in CpGV, CrleGV and EpapGV but in a different location compared to ChocGV.

### ClasGV-B is a new isolate from C. anastomosis

Previously, two betabaculoviruses have been sequenced from *Notodontidae*, ClanGV isolated from *C*. *anachoreta* and ClasGV-A from *C*. *anastomosis* [14, 19]. The viruses are cross infective and share 92% identity in their nucleotide sequence [14]. Both viruses contain 123 ORFs among which, 119 are homologous genes with high identities including 75 genes with more than 90% identity, and 31 from 80% to 89% [14]. However, sequence analysis showed that ClasGV-B is quite different from ClanGV and ClasGV-A. The nucleotide identity of ClasGV-B genome to that of ClanGV and ClasGV-A is 67% and 82%, respectively. The average amino acid identity of ClasGV-B ORFs to that of ClanGV and ClasGV-A is 51% and 52%, respectively. The gene arrangement (Fig 3) also showed that ClasGV-B is different from that of ClanGV and ClasGV-A. These results clearly indicated that ClasGV-B is different from ClanGV and ClasGV-A, and represents a new species of baculovirus isolated from *C*. *anastomosis*.

In summary, the analysis of the genome of ClasGV-B revealed that the virus is a new species of betabaculovirus isolated from *C*. *anastomosis*. Phylogenetically, it was most closely related to ErelGV. Typical baculovirus *hr*s were absent from the ClasGV-B genome but it contains two putative non-*hr* replication origins. The study contributes to the application of the virus as bioinsecticides and the elucidation of baculovirus evolution.

## Materials and Methods

### Viruses and viral DNA extraction

ClasGV-B was isolated from a *C*. *anastomosis* larva showing the typical features of baculovirus infection in a field in Hunan Province [18]. OBs were purified from infected larvae [62]. Extraction of viral genomic DNA from OBs was performed following the method reported previously [38]. Briefly, 100μl of OBs stock was washed three times with ddH_2_O and resuspended in 300 μl ddH_2_O. 20 μl proteinase K (20 mg/ml) was added and incubated with the sample at 37°C for 30 min, and then150 μl 3×Dissolving buffer (0.3M Na_2_CO_3_, 0.03M EDTA, 0.5M NaCl, pH 10.9) was added to release of ODVs from OBs at 37°C for 30 min. Afterwards, 15μl neutralization buffer (1M Tris, pH 7.0) and 50μl 10% SDS were added and incubated for another 30 min at 37°C. Viral DNA was extracted with phenol/chloroform and precipitated by ethanol. The DNA pellet was resuspended in 50μl TE and stored at 4°C.

### Sequencing and sequence analysis of the ClasGV-B genomic DNA

DNA was sequenced with the 454 Life Science platform (GS-FLX, Roche Applied Science) at the sequencing lab of the State Key Laboratory of Virology (Wuhan Institute of Virology, China). Newbler assembler of the GS-FLX Data Analysis Software was used for *de novo* assembly and regions with low quality data were refined by PCR amplification and Sanger sequencing. A total of 35,918 reads were obtained, with an average length of 333 bp, totally 11,950,277 nt, representing 110 coverage of the genome. The genomic DNA sequence was submitted to GenBank under the accession number KR091910.

Open reading frames (ORFs) were predicted using NCBI ORF finder (http://www.ncbi.nlm.nih.gov/gorf/gorf.html) [63] and FGENESV0 (http://linux1.softberry.com/berry.phtml) [64] by locating translation start and stop codons. ATG-initiated ORFs that are 150 nt or more with minimal overlap were selected for further analysis. Amino acid identities between homologous genes were carried out using the standard protein-protein BLAST algorithm from the results of protein database searches (http://blast.ncbi.nlm.nih.gov/Blast.cgi). Repeated sequences were searched following the method reported by Ferrelli *et al*. by using Blast2seq program from NCBI [10, 65]. The gene order of ClasGV-B with other baculoviruses was compared using gene parity plots [27].

### Phylogenetic analysis

Phylogenetic analysis was performed using the Maximum Likelihood method based on the JTT matrix-based model incorporated in the MEGA5 program [66]. Tree reliability was tested with bootstrap re-sampling using 1000 replicates. Concatomers of the 37 conserved core proteins from 73 baculovirus genomes were used to construct the phylogenetic tree.

## Supporting Information

S1 Table Characteristics of the ClasGV-B genome. Predicted ORFs are compared with homologues in seventeen betabaculoviruses, AcMNPV, NeseNPV and CuniNPV. The transcriptional orientation of each ORF is indicated by symbols: > (+ strand) and < (- strand). The presence of early (E) and late (L) promoter located within 150 nucleotides of the start codon is shown.
(XLS)
